# The Evolution in Electrochemical Performance of Honeycomb-Like Ni(OH)_2_ Derived from MOF Template with Morphology as a High-Performance Electrode Material for Supercapacitors

**DOI:** 10.3390/ma13214870

**Published:** 2020-10-30

**Authors:** Xiao Li, Jun Li, Ying Zhang, Peng Zhao, Ruyan Lei, Baige Yuan, Manman Xia

**Affiliations:** School of Materials Engineering, Shanghai University of Engineering Science, Shanghai 201620, China; xiaoli20201001@126.com (X.L.); yingzhang666666@163.com (Y.Z.); 17715145519@139.com (P.Z.); leiry564335@163.com (R.L.); ybg181213@163.com (B.Y.); 13783130648@163.com (M.X.)

**Keywords:** honeycomb-like structure, Ni-MOF, Ni(OH)_2_, electrode material, supercapacitor

## Abstract

Ni(OH)_2_ derived from an MOF template was synthesized as an electrode material for supercapacitors. The electrochemical performance of the electrode was adjusted by effectively regulating the morphology of Ni(OH)_2_. The evolution of electrochemical performance of the electrode with morphology of Ni(OH)_2_ was highlighted in detail, based on which honeycomb-like Ni(OH)_2_ was successfully synthesized, and endowed the electrode with outstanding electrochemical performance. For the three-electrode testing system, honeycomb-like Ni(OH)_2_ exhibited a very high specific capacitance (1865 F·g^−1^ at 1 A·g^−1^, 1550 F·g^−1^ at 5 mV·s^−1^). Moreover, it also presented an excellent rate capability and cycling stability, due to 59.46 % of the initial value (1 A·g^−1^) being retained at 10 A·g^−1^, and 172% of initial value (first circle at 50 mV·s^−1^) being retained after 20,000 cycles. With respect to the assembled hybrid supercapacitor, honeycomb-like Ni(OH)_2_ also displayed superior electrochemical performance, with a high energy density (83.9 Wh·kg^−1^ at a power density of 374.8 W·kg^−1^). The outstanding electrochemical performance of Ni(OH)_2_ should be attributed to its unique honeycomb-like structure, with a very high specific surface area, which greatly accelerates the transformation and diffusion of active ions.

## 1. Introduction

Metal-organic framework (MOF), refers to a crystalline porous material with a periodic network structure, formed by the self-assembling of metal ions and organic ligands [1]. MOF has been widely applied in separation [2], catalysis [3], sensing [4], ion conduction [5], etc., due to its high porosity, large specific surface area, and structural diversity [6]. Recently, MOF has attracted extensive attention in the energy storage field, and has been proved to be a high-performance electrode material for supercapacitors [7]. Gao et al. [8] prepared a 3D waxberry-like Ni-MOF for supercapacitors. The resultant electrode displayed a specific capacitance of 781.2 F·g^−1^ at a current density of 1 A·g^−1^, and retained 60% of its initial value at 10 A·g^−1^. Moreover, about 45% of its initial specific capacitance was retained after 5000 cycles at 15 A·g^−1^. Du et al. [9] fabricated a cage-like Co-MOF and analyzed its electrochemical behavior in KOH solution. The substance demonstrated a specific capacitance of 236.2 F·g^−1^ at 1 A·g^−1^ and an outstanding cycling stability, with a specific capacitance retention of 64.04% after the cycle numbers increased to 3000. Sundriyal et al. [10] synthesized a 3D cubic-like Zn-MOF. The specific capacitance reached 317.6 F·g^−1^ at the scanning rate of 1 mV·s^−1^, and a high capacitance retention of 99% over 1000 cycles was obtained. However, MOF as an electrode material still has many problems to be solved. MOF usually has a poor conductivity [11] owing to the portion of organic substances with a low conductivity involved in it, which is not beneficial to the transportation of active ions within it. Moreover, the structure of MOF is not stable [12], resulting from the weak binding force of the coordination bond, which may cause relatively poor cycling stability during repeated charging and discharging. Moreover, the specific capacitance of MOF is comparatively low; of approximately 500–800 F·g^−1^ at the current density of 1 A·g^−1^ [13,14,15].

Recently, a novel method had been proposed to solve these weaknesses, namely MOF as a precursor or template is transformed into a new derivative (hydroxides). The derivative presents a more outstanding electrochemical performance than MOF, due to its higher specific capacitance and original framework. Tang et al. [16] synthesized sheet-like Ni-MOF and sphere-like Ni-MOF by the hydrothermal method, then transformed them into Ni(OH)_2_ with similar morphology by immersing them in 2 M KOH for 5 h. Sphere-like Ni(OH)_2_ possessed the larger specific capacitance of 982 F·g^−1^ at 1 A·g^−1^ and higher retention of 56% after the current density increased to 10 A·g^−1^ when compared with sheet-like Ni(OH)_2_ (861 F·g^−1^ and 25% retention). Moreover, its specific capacitance only dropped by about 6% after 1000 cycles, which was far less than that of sheet-like Ni(OH)_2_ (about 15% loss). Zhang et al. [17] first prepared rod-like Ni-MOF crystals by the hydrothermal method, then successfully fabricated Ni(OH)_2_ with different morphologies by immersing Ni-MOF in 6 M KOH for 6 h at different temperatures (30 °C, 60 °C, 75 °C, 90 °C, and boiling). The results indicated that Ni(OH)_2_ prepared at 75 °C exhibited the highest specific capacitance of 713.2 C·g^−1^ at 1 A·g^−1^, excellent rate capability (58.2% retention at 10 A·g^−1^), and outstanding cycling stability (65% of its initial capacitance retained after 400 cycles). Wang et al. [18] fabricated a porous-like Co(OH)_2_ using Co-MOF as the template by a solid-solid transformation method. The Co(OH)_2_ electrode materials exhibited an outstanding specific capacitance of 604.5 F·g^−1^ at 0.1 A·g^−1^. Moreover, the product also had excellent rate capability (454.6 F·g^−1^ at 5 A·g^−1^) and retained 84.5% of its original specific capacitance after 2000 cycles at 2 A·g^−1^. Based on the previous investigations into hydroxides synthesized with MOF, as a precursor or template, it can be concluded that electrochemical performance is closely associated with morphology. The morphology strongly depends on the initial morphology of the MOF and the synthesized conditions of hydroxides, with respect to the heating time, temperature, and the selection of soak solution, etc. However, there are few reports about the evolution of the morphology of MOF-derived hydroxides with concentration of soak solution.

This work synthesized sphere-like Ni-MOF by the hydrothermal method, then transformed it into Ni(OH)_2_ by immersing it in different concentrations of KOH (2 M, 6 M, and 8 M) for 6 h at 75 °C. The effects of the concentrations of Ni(OH)_2_ on the morphology structure and electrochemical performance were investigated comprehensively, based on which, the relationship among them was established. The optimal concentration of Ni(OH)_2_ was determined, in which honeycomb-like Ni(OH)_2_ was successfully fabricated and demonstrated the best electrochemical performance.

## 2. Materials Procedures

### 2.1. Synthesis of Ni-MOF

Nickel nitrate hexahydrate (Ni(NO_3_)_2_·6H_2_O, 98.5%, Shanghai Titan Scientific Co., Ltd., Shanghai, China), 1,3,5-benzenetricarboxylic acid (PTA, 99%, Aladdin Industrial Corporation, Shanghai, China), and N,N-Dimethylformamide (DMF, ≥99.9%, Aladdin Industrial Corporation) were used to produce Ni-MOF. Ni(NO_3_)_2_·6H_2_O (4 mmol, 1.162 g) and PTA (1 mmol, 0.166 g) were separately dissolved in DMF (15 mL) with stirring for about 30 min at room temperature. Then, the two solutions were put together and stirred for 30 min. Finally, the mixed solution was placed into a Teflon-lined stainless steel autoclave to heat at 150 °C for 12 h. The received product was separately centrifuged with DMF and ethanol three times before vacuum drying 60 °C overnight.

### 2.2. Synthesis of Ni(OH)_2_ with Ni-MOF as the Template

0.5 g Ni-MOF was immersed in 200 mL KOH solution with different concentrations of 2 M, 6 M, and 8 M, and stirred at 75 °C for 6 h. The received product was separately centrifuged with the deionized water and ethanol three times, and dried at 60 °C for 12 h. The synthesized Ni(OH)_2_ at 2 M, 6 M, and 8 M KOH solutions were named as 2 M-Ni(OH)_2_, 6 M-Ni(OH)_2_, 8 M-Ni(OH)_2_, respectively.

### 2.3. Preparation of the Working Electrode

Nickel foam was selected as the current collector (1.2 × 1.5 cm, Shanxi Power Source Battery Materials Co., Taiyuan, China), which was subsequently cleaned in acetone, 1 M HCl, ethanol, and deionized water for 10 min to remove the organic matter and oxide layer on it, then dried at room temperature overnight. The active substances (80 wt.%), acetylene black (10 wt.%) and poly vinylidene fluoride (PVDF, 10 wt.%), were mixed into the N-methyl-2-pyrrolidone (NMP) solvent. The formed slurry was uniformly coated on nickel foam, with a mold with a fixed area (1 cm × 1 cm), to ensure the weight of active substance of about 2 mg. Then the coated part was folded and pressed under a pressure of 10 MPa for 10 s. Finally, the prepared working electrode was dried at 120 °C for 4 h and soaked in KOH solution overnight for electrochemical tests. The saturated calomel electrode (SCE) and a graphite sheet (20 mm × 25 mm) were used as the reference electrode (RE) and the counter electrode (CE), respectively. A 6 M KOH solution was chosen as the electrolyte. Meanwhile, the activated carbon (AC) electrode in the two-electrode testing system was fabricated by mixing the 90 wt.% active carbon and 10 wt.% PVDF in the NMP solution, the following process of preparing the AC electrode was the same as above.

### 2.4. Structural Characterization

Phase constituents of all samples were analyzed by an X-ray diffractometer (XRD, D2-PHASER Bruker, Karlsruhe, Germany) using Cu-Kα radiation (γ = 0.1540560 nm). The morphology and structure of the samples were observed by a field-emission scanning electron microscope (FESEM, S-4800, Hitachi, Tokyo, Japan) and a transmission electron microscope (TEM, JEM-2100F, JEOL, Tokyo, Japan) equipped with energy dispersive spectrometer (EDS), respectively. Chemical compositions and their chemical valences were determined using an X-ray photoelectron spectroscope (XPS, Thermo Fisher Scientific, Waltham, MA, USA) equipped with a monochromatic Al Kα X-ray source. The specific surface area and mesopore size distribution of the samples were analyzed by an automatic surface area and porosity analyzer (ASAP-2460, Micromeritics, Shanghai, China), and calculated by the Brunauer–Emmett–Teller (BET) and Barrett–Joyner–Halenda (BJH) methods, respectively. Characteristic functional groups were analyzed by a Fourier transform infrared spectrometer (FTIR, Nicolet, Shanghai, China) in a range of 4000–400 cm^−1^.

### 2.5. Electrochemical Measurement

Electrochemical performance of the electrodes was tested by cyclic voltammetry (CV), galvanostatic charge-discharge tests (GCD), and electrochemical impedance spectroscopy (EIS) on an electrochemical workstation (CHI 760E, CH Instrument Inc, Shanghai, China). The potential window was set as 0–0.5 V for Ni-MOF and −0.1–0.6 V for Ni(OH)_2_, and the scanning rate was ranged from 5 to 50 mV·s^−1^ in the CV tests. The GCD tests were conducted with the potential between 0 and 0.33 V, and the range at 1, 2, 3, 4, 6, 8, and 10 A·g^−1^. The cycling stability was measured according to the CV tests for 20,000 cycles at 50 mV·s^−1^. The EIS tests were carried out in the frequency range from 0.1 MHz to 0.01 Hz.

## 3. Results and Discussions

### 3.1. Structure Characterization

**Figure 1:**
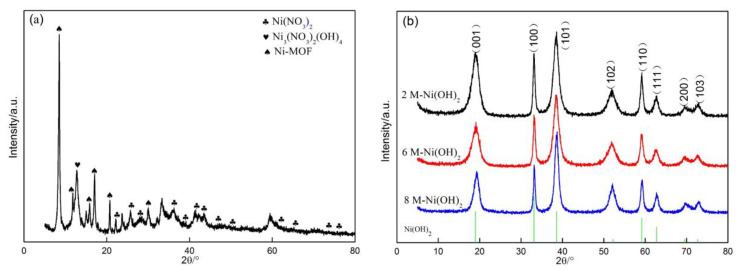
XRD patterns of (**a**) Ni-MOF and (**b**) Ni(OH)_2_.

Figure 1a shows the XRD patterns of the as-prepared sample prior to the immersion in the KOH solution. Based on the reported research about Ni-MOF [19] by Yue et al., it can be confirmed that Ni-MOF was successfully fabricated. Especially the two characteristic peaks related to Ni-MOF at 8.33° and 17.12° provide strong evidence for the existence of Ni-MOF [20]. However, many unexpected peaks were also clearly observed; the indexed results confirmed the existence of Ni_3_(NO_3_)_2_(OH)_4_ and Ni(NO_3_)_2_. The two inert substances may have produce a negative effect on electrochemical performance of the Ni-MOF. Therefore, Ni-MOF samples subjected to the treatment in the KOH solution were sufficiently washed by ethanol and deionized water to remove the impurities.

Figure 1b indicates the XRD patterns of the samples treated in the KOH solution with different concentrations (2 M, 6 M, and 8 M). These patterns are very similar, in which eight sharp diffraction peaks can be clearly observed at 18.98°, 33.15°, 38.61°, 52.23°, 59.17°, 62.72°, 69.58°, and 72.67°. The indexed results indicate that their d values are well in accordance with those of Ni(OH)_2_ (JCPDS, No. 00-001-1047). This implies that highly purified Ni(OH)_2_ was successfully synthesized. A close examination reveals an interesting phenomenon, namely the peak width presents a downward tendency with the increase in KOH concentration. This change should be attributed to the gradual coarsening of Ni(OH)_2_ with increase of the KOH concentration. It is well known that the growth of Ni(OH)_2_ will be seriously inhibited in a low concentration of KOH solution due to the diffusion control of KOH, resulting in fine Ni(OH)_2_ being obtained. The diffusion of KOH as a key growth rate-controlling step of Ni(OH)_2_ gradually attenuates with the increase in KOH concentration, so that Ni(OH)_2_ tends to grow into coarse grains.

**Figure 2:**
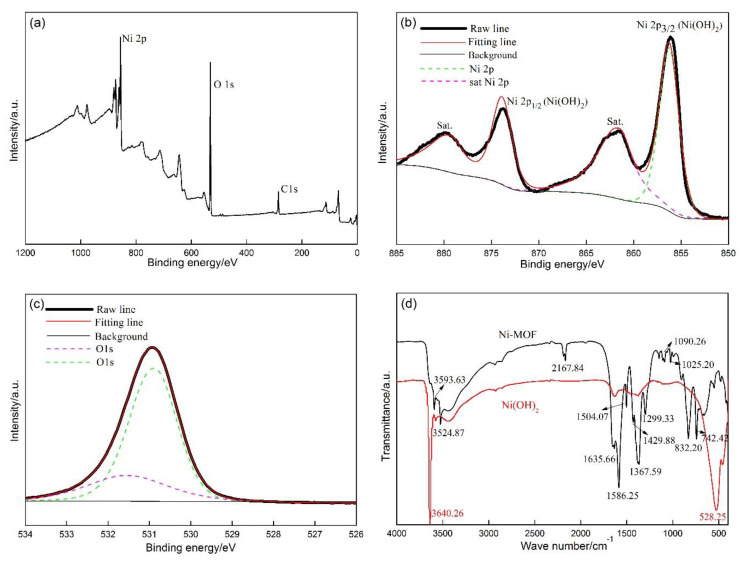
(**a**) Survey XPS spectrum, high resolution spectra of Ni 2p (**b**), O 1s (**c**) of the Ni(OH)_2_, (**d**) FTIR spectra of Ni-MOF and Ni(OH)_2_.

XPS was employed to detect the chemical valences of the elements involved in the sample (Figure 2a). The sample was mainly composed of Ni, O, and a small amount of C (Figure 2a). Four peaks can be fitted in the Ni 2p high-resolution spectrum (Figure 2b). The two peaks with the binding energies of 855.31 eV and 873.21 eV are designated to Ni^2+^ in Ni(OH)_2_, accompanied by two corresponding satellite peaks related to Ni(OH)_2_, which are observed at 879.13 eV and 861.03 eV [21]. With regard to the O 1s high-resolution spectrum (Figure 2c), the strong peak at 530.93 eV implies the existence of OH^−^ in Ni(OH)_2_ [22]. The XPS result further confirm that Ni-MOF is successfully transformed into Ni(OH)_2_. Figure 2d shows the FT-IR spectra of the as-synthesized samples. The adsorption bands at 3593.63 cm^−1^ and 3524.87 cm^−1^ for Ni-MOF could be ascribed to the stretching vibration of water molecules, indicating the existence of H_2_O molecules in Ni-MOF [23]. The peaks at 2167.84 cm^−1^ were caused by the vibration of the C=O, implying that carbon dioxide in the air was absorbed by the samples [24]. The adsorption band at 1635.66 cm^−1^ derived from the stretching vibration of the carbon–carbon double bond (C=C) [25], meaning that the organic substance (PTA) applied for the fabrication of Ni-MOF was not completely removed. Moreover, the characteristic adsorption bands at 1504.07 cm^−1^, 1586.25 cm^−1^, 1429.88 cm^−1^, and 1367.59 cm^−1^ are ascribed to the symmetrical and asymmetrical stretching vibration of COO^−^, confirming that COO^−^ and Ni^2+^ in Ni-MOF is bonded in the two ways [26]. Furthermore, the bands at 1299.33 cm^−1^, 1090.26 cm^−1^, and 1025.20 cm^−1^ could be associated with the symmetrical and asymmetrical stretching vibration of NO_3_^-^, verifying the residual substance of Ni(NO_3_)_2_, as demonstrated in the XRD results. The observed peaks around 832.20 cm^−1^, and 742.42 cm^−1^ originated from the stretching vibration of the aromatic [27], owing to the PTA ligands. After the sample was immersed in the KOH solution, the peaks related to residual PTA, Ni(NO)_3_, and carboxyl organic ligands in Ni-MOF almost vanished, accompanied by which two new peaks were observed at 3640.26 cm^−1^ and 528.25 cm^−1^. They were assigned to the stretching vibration of OH groups and Ni-O-H, confirming the formation of Ni(OH)_2_ [28].

**Figure 3:**
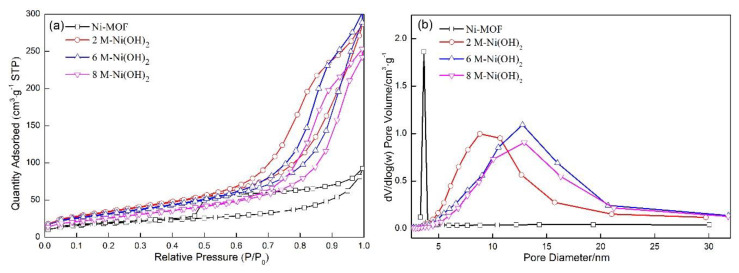
(**a**) N_2_ adsorption–desorption isotherm of the Ni-MOF and Ni(OH)_2_, (**b**) the corresponding pore size distribution curves of Ni-MOF and Ni(OH)_2_.

Figure 3a indicates the N_2_ adsorption/desorption isotherm curves of the Ni-MOF and Ni(OH)_2_ samples. The hysteresis loop formed in the curve related to Ni-MOF can be classified as type H_1_, which reveals that the distribution in pore diameter is comparatively narrow in Ni-MOF. However, the type of hysteresis loop is transformed from type H_1_ into type H_3_ after immersion in the KOH solution, indicating that the pores mainly originate from the gaps among a large number of agglomerated sheet-like particles. The Barrette–Joyner–Halenda pore size distribution is shown in Figure 3b, based on which the specific surface area, total pore volume, and average pore size can be calculated (Table 1). The distribution in pore diameter of Ni-MOF was very narrow, and was mainly in the range from 3.5 nm to 3.8 nm, with a volume of 0.075 m^3^·g^−1^ (about 61.98% of the total volume). The average pore diameter and the total pore volume were also the lowest of about 8.55 nm and 0.121 cm^3^·g^−1^ among all samples, as shown in Table 1. The pores with a wider distribution of 5–20 nm were formed due to the curves moving toward the right and becoming wider after immersion in the KOH solution, accompanied by the average pore size and the total pore volume of Ni(OH)_2_ increasing to about 14.87 nm and 0.431 cm^3^·g^−1^ (average value). With respect to the immersed samples, the increase in KOH concentration caused the decrease in total pore volume (0.447 cm^3^·g^−1^ for 2 M-Ni(OH)_2_), however the average pore diameter was enlarged (13.62 nm for 2 M-Ni(OH)_2_). The above changes produced a significant effect on the specific surface area. The specific surface area of Ni-MOF (67 m^2^·g^−1^) drastically increased by about 94% (130 m^2^·g^−1^) when subject to the immersion in a 2 M KOH solution. However, the value was gradually reduced to 118 m^2^·g^−1^ (6 M-Ni(OH)_2_) and 98 m^2^·g^−1^ (8 M-Ni(OH)_2_) with the increase in concentration of KOH. The above changes are mainly related to the structural evolution of the samples, before and after the immersion in the KOH solution with different concentrations. When Ni-MOF was subjected to the treatment in the KOH solution, the organic bridges with a large size were replaced by the small hydroxide ions [29], which caused a great reduction of the lattice volume. A large number of particles suffered from a serious contraction, among which numerous new micropores were produced, and the initial tiny pores were enlarged into coarse pores. As a result, the average pore diameter and total pore volume were correspondingly increased. Moreover, the increase in total pore volume and the decrease in weight made the specific surface area greatly enhanced.

A further increase in KOH concentration will accelerate the growth of Ni(OH)_2_, causing the pores to be compressed in volume. The specific surface area is correspondingly reduced. However, the average pore diameter demonstrates a slight increase, which is in connection with change in pores with different diameters. Along with the increase in concentration of KOH, the growth rate of the nanosheets will be correspondingly enhanced. As a result, tiny pores among Ni(OH)_2_ nanosheets may be merged and disappear completely, accompanied with which the comparatively large pores may be transformed into tiny pores. These two changes will cause a reduction in pore volume with the increase in concentration of KOH. However, the two changes will result in a contrary change in average pore diameter. The disappearance of tiny pores will cause an increase in average pore diameter, however, the compression of large pores into tiny pores will cause the decrease in average pore diameter. With the increase in concentration of KOH, the disappearance of tiny pores may play the dominant role in the change of average pore diameter when compared with the compression of large pores. Consequently, the average pore diameter is increased with the KOH concentration enhanced from 6 M to 8 M. The high specific surface area in the 2 M-Ni(OH)_2_ sample provided active sites for electrode reactions, which contributed to the improvement in electrochemical performance. Moreover, the comparatively small pores retarded the rapid penetration of the electrolyte into the inner of the spherical nanoparticles, which played a negative role in electrochemical performance. Therefore, the three modified samples were employed for electrochemical tests with the unmodified sample as a reference. Then, the optimum KOH concentration was confirmed. The total pore volume was substantially increased by about 266% (0.443 m^3^·g^−1^), and the pore distribution of pore diameter was greatly enlarged, from 3.50 nm to about 20.13 nm. The average pore diameter was increased to 13.62 nm. With the further increase in concentration of KOH, the curves moved toward the right, indicating that the volume percentage of the small pores was reduced. 

The specific surface area was about 67 m^2^·g^−1^ in Ni-MOF, increased by about 94% (130 m^2^·g^−1^). However, the value was gradually reduced to 118 m^2^·g^−1^ (6 M-Ni(OH)_2_) and 98 m^2^·g^−1^ (8 M-Ni(OH)_2_) with the increase in concentration of KOH. A similar change could be observed in the total volume after the immersion in the 2 M KOH solution, and then slightly reduced with increasing the KOH concentration. Average pore diameter was also increased at first (about 59%), however, the tendency was maintained with the increase in KOH concentration. 

### 3.2. Morphology Characterization

**Figure 4:**
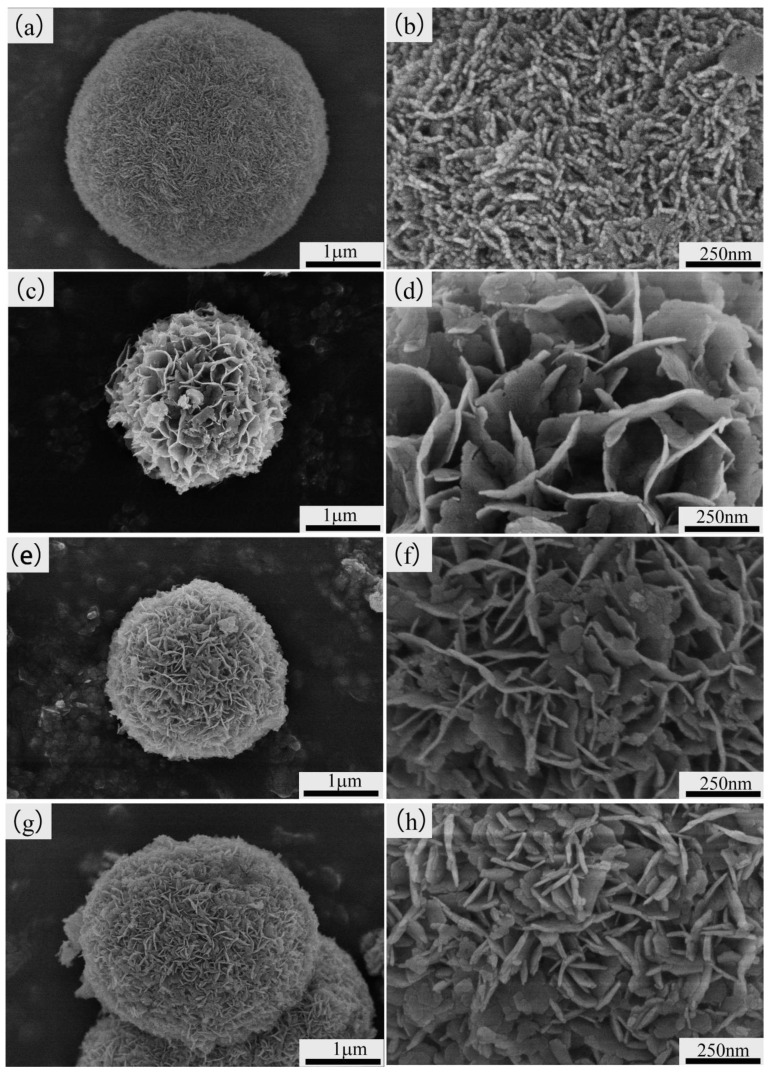
Field-emission scanning electron microscope (FE-SEM) images of Ni-MOF and Ni(OH)_2_ samples. (**a**,**b**) Ni-MOF, (**c**,**d**) 2 M-Ni(OH)_2_, (**e**,**f**) 6 M-Ni(OH)_2_, and (**g**,**h**) 8 M-Ni(OH)_2_, respectively.

**Figure 5:**
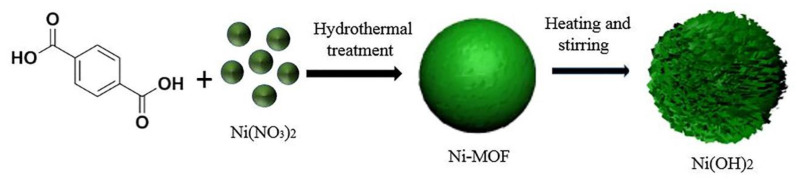
Schematic illustration of the fabrication mechanism of Ni(OH)_2_.

Figure 4 shows the morphology of the as-synthesized samples. Ni-MOF was composed of the spherical particles with a diameter of about 3.6 µm (Figure 4a), in which a large number of finer worm-like particles can be observed (Figure 4b). A similar structure was also reported by Du et al. [30]. After soaking in 200 mL aqueous solution of KOH and stirring at 75 °C for 6 h, the morphology of the spherical particles changed greatly due to the worm-like particles being transformed into fine nanosheets, with a thickness of about 38 nm (Figure 4d). The honeycomb structure was obtained on the surfaces of spherical particles due to those nanosheets clustering together, in which numerous channels could be formed. This means that the transformation from Ni-MOF into Ni(OH)_2_ could have endowed the sample with a higher specific surface area. The formation of the sheet-like Ni(OH)_2_ can be clearly discerned from the schematic illustration (Figure 5). This is supposed to follow a “crystal-crystal conversion” mechanism. The carboxyl organic ligands of Ni-MOF are replaced by excessive amounts of OH^−^ when Ni-MOF is immersed in KOH solution. Ni(OH)_2_ belongs to the hexagonal structure, in which the nickel atoms and oxygen atoms are arranged in a layered structure connected by Vander Waals forces. Ni(OH)_2_ molecules are located on the crystal plane parallel to the Z axis, and tend to grow into a sheet shape along the X-Y face. The nanosheets per unit area were correspondingly increased in volume fraction when the concentration of KOH increased to 6 M and 8 M, causing the channels among the nanosheets to be greatly reduced in size. That is to say, the increasing concentration of KOH promoted the growth of the nanosheet and made the spherical particles become denser.

**Figure 6:**
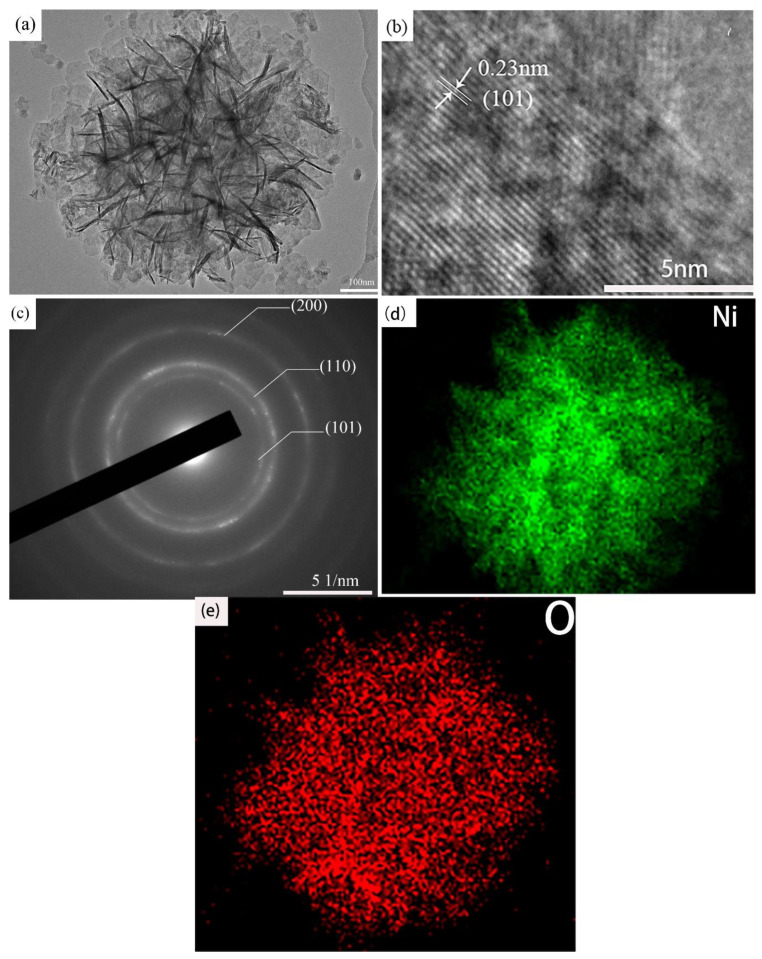
(**a**) TEM images of the 6 M-Ni(OH)_2_, (**b**) high resolution TEM (HRTEM), (**c**) selected area electron diffraction (SAED) and EDS mapping of (**d**) Ni and (**e**) O.

Figure 6 displays the microstructure characteristics of the as-prepared samples. It is clear that 6 M-Ni(OH)_2_ demonstrates an irregular sphere-like shape, with a diameter of about 737 nm (Figure 6a). Numerous fine nanosheets, with a thickness of about 10 nm and involved in the comparatively coarse sphere-like particle, are crisscrossed into a honeycomb structure, among which tiny gaps can be formed. These gaps not only provide abundant channels to promote the diffusion of active hydroxyl ions into the inner of the spherical 6 M-Ni(OH)_2_ particles, but also allow them to be closely in contact with more nanosheets. These two factors produce the synergistical effect and greatly enhance the electrochemical performance. Figure 6b displays the lattice fringes of the samples. The d-value was determined as about 0.23 nm, which is consistent with that of the (101) plane in Ni(OH)_2_. Figure 6c shows the relevant SAED (elective area electron diffraction) pattern, in which some diffraction circles with different radius can be observed. The indexed results indicate that they correspond to (101), (110), and (200) planes of Ni(OH)_2_ (JCDPS: No. 00-001-1047). The element mapping of O and Ni (Figure 6d,e) shows that they are uniformly distributed in the particles.

### 3.3. Electrochemical Measurements of Ni-MOF and Ni(OH)_2_ Samples

**Figure 7:**
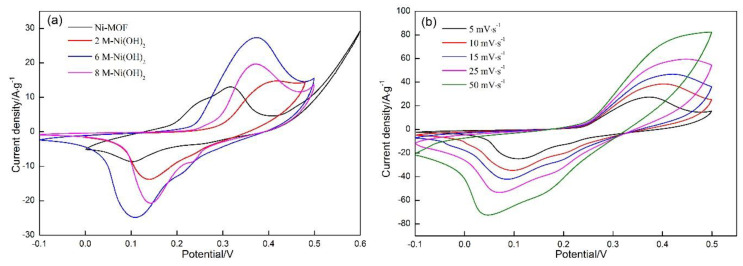
Cyclic voltammetry (CV) curves of the as-prepared samples. (**a**) Ni-MOF and Ni(OH)_2_ at the scanning rate of 5 mV·s^−1^; (**b**) 6 M-Ni(OH)_2_ at different scanning rates.

Figure 7a demonstrates the CV curves of Ni-MOF and Ni(OH)_2_ samples synthesized in different concentrations of KOH solution at the scanning rate of 5 mV·s^−1^. An oxidation peak can be clearly observed at approximately 0.32 V for Ni-MOF, coupled with a corresponding reduction peak appearing at about 0.1 V. The redox peaks correspond to the following reversible reaction [31]:

(1) $$[{\mathrm{Ni}}_{3}{\left(\mathrm{OH}\right)}_{2}{\left(C_{8}H_{4}O_{4}\right)}_{2}\cdot \left(H_{2}O{)}_{4}\right]\cdot 2H_{2}O+{\mathrm{OH}}^{-}-e^{-}\Leftrightarrow \;\left[{\mathrm{Ni}}_{3}O\left(\mathrm{OH}\right){\left(C_{8}H_{4}O_{4}\right)}_{2}\cdot (H_{2}O{)}_{4}\right]\cdot 2H_{2}O+H_{2}O$$

Referring to the Ni(OH)_2_ samples, a pair of redox peaks can also be observed at 0.12 V and 0.4 V. The difference in peak potential between Ni-MOF and Ni(OH)_2_ implies that the redox reactions occurring on the Ni(OH)_2_ electrodes are different from reaction (1), which can be described as follows [32]:

(2) $$\mathrm{Ni}{\left(\mathrm{OH}\right)}_{2}+{\mathrm{OH}}^{-}\Leftrightarrow \;\mathrm{NiOOH}+H_{2}O+e^{-}\;$$

For the given mass of active substances, scanning rate and potential window, the integrated area surrounded by the CV curves can be applied to characterize the specific capacitance of the electrodes, between which a proportional relationship can be established as follows [33]:

(3) $$C=\frac{1}{mv\left(\Delta V\right)}\int I\left(V\right)\mathrm{dV}$$

where *m* (g) is the mass of active material, *v* (mV/s) represents the scanning rate, Δ*V* signifies the potential window (*V*), and *I* (A) denotes the instant current.

It is clear that the integrated area of 2 M-Ni(OH)_2_ is significantly larger than Ni-MOF, which indicates that a higher specific capacitance can be obtained by transforming Ni-MOF into Ni(OH)_2._ Some research reported that Ni(OH)_2_ exhibits a higher specific capacitance than Ni-MOF. As far as Ni(OH)_2_ is concerned, it can be concluded that 6 M-Ni(OH)_2_ demonstrated the highest specific capacitance, followed by 2 M-Ni(OH)_2_, and 8 M-Ni(OH)_2_. The differences among their morphologies were likely responsible for the change. As analyzed above, a relatively high specific surface area was acquired in 6 M-Ni(OH)_2_ (118 m^2^·g^−1^), among the three Ni(OH)_2_ samples. High specific surface area allows more active ions to sufficiently contact active substances. On the other hand, the transportation paths for active ions and electrons are greatly reduced. Moreover, a large number of pores involved in the spherical particles will provide more tunnels for facilitating active ions’ diffusion into the interior of spherical particles. These three factors will produce the synergistic effect, which results in more active ions participating in the reactions on the electrodes. Based on the Equation (3), their specific capacitance can be calculated precisely (Table 2). The value of 6 M-Ni(OH)_2_ reached 1550 F·g^−1^, which is an increase of about 106%, when compared with that of Ni-MOF.

**Figure 8:**
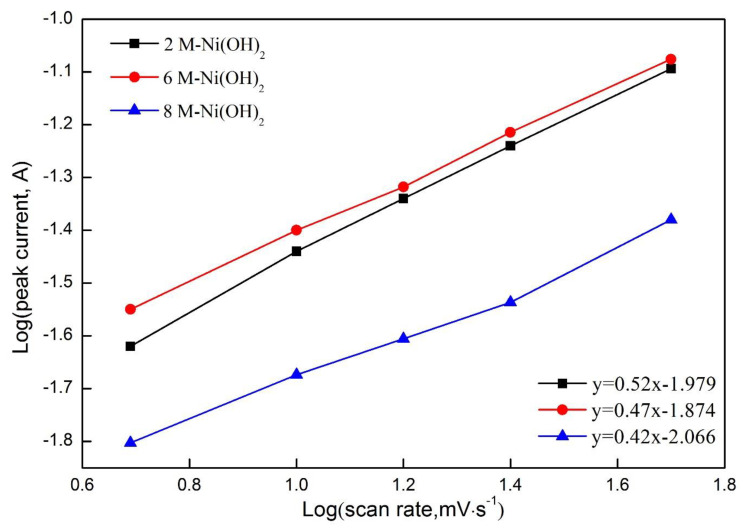
Logarithmic relationship of different concentrations of Ni(OH)_2_ samples.

Figure 7b displays the CV curves of 6 M-Ni(OH)_2_ samples at scanning rates ranging from 5 mV·s^−1^ to 50 mV·s^−1^ in a 6 M KOH solution. It is also worth noticing that the oxidation peaks shifted toward the higher potential with the increase in scanning rate, accompanied with a converse shift of the reduction peaks. This phenomenon is associated with the change in the electrode surface. The reactions occurring on the electrode surface can be regarded as the quasi-equilibrium state at a comparatively low scanning rate, which are not subjected to the diffusion control. The active ions around the electrode surface are drastically consumed with the increasing scanning rate, resulting in the reactions being controlled by the diffusion of active ions, and gradually deviating from the equilibrium state. Consequently, the oxidation and reduction peaks shift toward the opposite direction. The rate capability of the electrode can be evaluated by the change in specific capacitance obtained at different scanning rates. As shown in Table 3, the specific capacitance presents a downward tendency with the increase in scanning rate, due to the reactions being gradually controlled by the diffusion of active ions. As shown in Figure 8, the peak current presents a linear relationship with the scanning rate, the obtained slopes are around 0.5, which indicates that the reactions occurring on the electrodes belonged to the typical diffusion-controlled process. When the scanning rate was increased from 5 mV·s^−1^ to 50 mV·s^−1^, the specific capacitance retained 38.5% of that obtained at 5 mV·s^−1^. This indicates that the electrode material demonstrated excellent rate capability.

**Figure 9:**
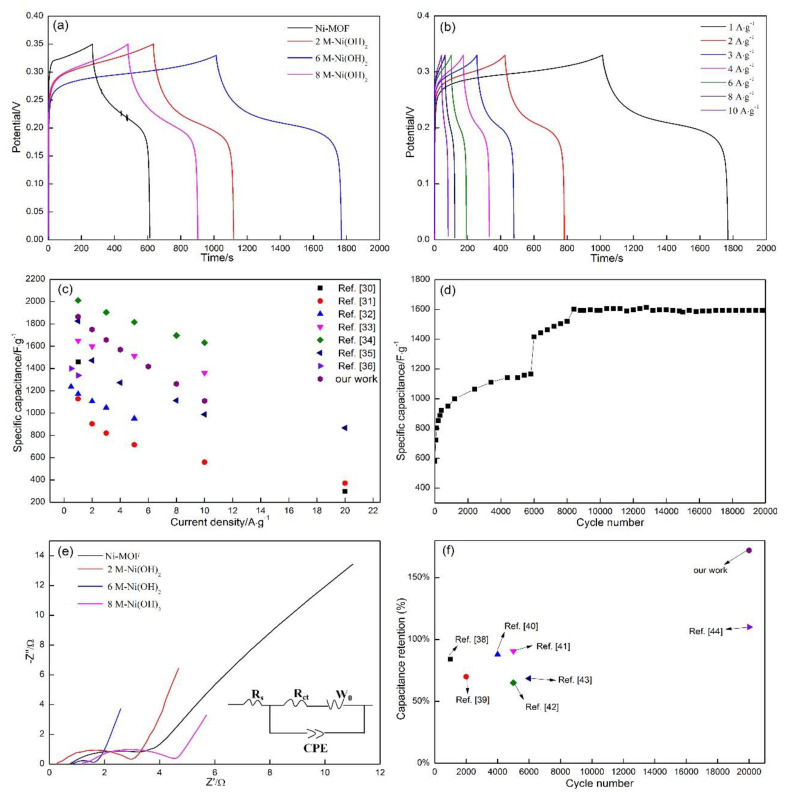
(**a**) Ni-MOF and Ni(OH)_2_ at the current density of 1 A·g^−1^. (**b**) 6 M-Ni(OH)_2_ at different current densities. (**c**) The comparison between the specific capacitance obtained in our work and those reported in other related references. (**d**) Cycling tests for 6 M-Ni(OH)_2_ at the scanning rate of 50 mV·s^−1^ up to 20,000 cycles. (**e**) Nyquist plots of Ni-MOF and Ni(OH)_2_ samples. (The inset is the equivalent circuit). (**f**) The comparison between the capacitance retention obtained in our work and those reported.

The galvanostatic charge–discharge technique was applied to further determine the specific capacitance of Ni-MOF and Ni(OH)_2_ (Figure 9a). The charge–discharge platforms resulting from the redox reactions can be detected, in which the duration of Ni(OH)_2_ is longer than that of Ni-MOF. Referring to Ni(OH)_2_, the duration of 6 M-Ni(OH)_2_ was the longest, followed by that of 2 M-Ni(OH)_2_ and 8 M-Ni(OH)_2_. This indicates that the 6 M-Ni(OH)_2_ possessed the highest specific capacitance. The specific capacitance of all samples can be calculated by the following formula [34]:

(4) $$C_{m}=\frac{I\Delta t}{m\Delta V}$$

where *C_m_* (F·g^−1^) is the specific capacitance, *I* (A) represents the constant discharge current, *m* (g) is the mass of the sample, and Δ*V* (V) is the potential window, while Δ*t* represents the discharge time. 

As shown in Table 4, the specific capacitance of 6 M-Ni(OH)_2_ is 1865 F·g^−1^ at the current density of 1 A·g^−1^, which is enhanced by approximately 195% when compared with that of Ni-MOF. Followed by 2 M-Ni(OH)_2_ (1380 F·g^−1^), and 8 M-Ni(OH)_2_ (989 F·g^−1^), respectively. 

Figure 9b displays the GCD curves of 6 M-Ni(OH)_2_ with the current densities ranging from 1 A·g^−1^ to 10 A·g^−1^. The specific capacitance is reduced from 1865 F·g^−1^ to 1109 F·g^−1^, about 59.46% of the initial value maintained. The specific capacitance of the 6 M-Ni(OH)_2_ acquired by galvanostatic charge–discharge was further compared with other related literature reports (Figure 9c) [35,36,37,38,39,40,41]. It can be noted that the specific capacitances obtained at the given current density in our work are situated at a comparatively high level among those reported values. 

Figure 9d shows the change in specific capacitance with the cycle number. The curve demonstrates an upward tendency, with the cycle number increased to about 8400 cycles, and then maintains a comparatively stable value up to 20,000 cycles. The initial increase in retention for the electrode material subject to 8400 cycles may be related to the continuous activation of active substances. The redox reactions mainly occur on the surface of an active substance (referring to the nanosheet surfaces located at the outer of the spherical particles) at the initial cycling, indicating that only a partial part of the substances were utilized. Active ions gradually diffuse into the interior of the spherical particle and participate in the reactions with more nanosheets, with the increase of cycle numbers, accompanied with which the specific capacitance is correspondingly enhanced. When the cycle number was increased to 8400, the active substances were sufficiently utilized, causing the specific capacitance to be retained at a comparatively stable level. The value is only a slight reduction of about 0.5%, which indicates that the active substances could maintain an excellent structural integrity, free of deboning and pulverization, and possessed a good activeness. This phenomenon has also been observed in similar studies. Zhang et al. [32] prepared Ni-MOF derived NiO/C nanospheres on reduced graphene oxide and fabricated an HSC device with the NiO/C/rGO and SCC as the electrode materials. The results of cyclic stability showed that the samples retained about 120% of their original specific capacitance at the current density of 2 A·g^−1^ after 3000 cycles. Tian et al. [42] successfully synthesized PPNF@MOF as efficient supercapacitor electrode material. The specific capacitance presented an upward trend during the first 1000 cycles in the cycling testing. Ranjbar et al. [43] successfully synthesized Ni/Co-based metal organic frameworks (MOFs) as an advanced electrode material for supercapacitors. The results of cycling life also showed that the retention rate displayed a slightly increase in the first 5000 cycles. This phenomenon is usually ascribed to the “activation” process during the cycling. 

Figure 9e displays the electrochemical impedance spectroscopy (EIS) curves of Ni-MOF and Ni(OH)_2_ samples at frequencies ranging from 10^5^ to 0.01 Hz in a 6 M KOH electrolyte (inset displays the fitting circuit). The Nyquist plots are composed of an obvious semicircle in the high frequency region and a straight line in the low frequency. The intercept of the semicircle at the real axis (Z’) can be applied to characterize Rs (ohmic resistance) from the electrolyte, electrode material, and some other possible ohmic resistances between the working electrode and the reference electrode. As shown in Table 5, there are hardly any differences between the two values obtained in Ni-MOF and 6 M-Ni(OH)_2_. Rct, denoted as the charge transfer resistance, is relevant to the diameter of the semicircle, which originates from the redox reactions occurring between the electrode and the electrolyte. The value of 6 M-Ni(OH)_2_ is greatly reduced, by about 74% (0.72 Ω), when compared with that of Ni-MOF (2.75 Ω), indicating that the resistance to redox reactions was significantly decreased. This should be attributed to the great reduction in the transportation path of active ions resulting from the increase in specific surface area. W_0_ demonstrates the Warburg impedance [42], which is connected with the slope of the straight line. This value can be used to characterize the diffusion rate of active ions inside the electrode materials. The value of 6 M-Ni(OH)_2_ (0.86 Ω) is far smaller than that of Ni-MOF (2.549 Ω), which should be ascribed to the synergistic effect of high specific surface, reduced transportation paths, and large number of pores in 6 M-Ni(OH)_2_ samples, as illustrated in the previous analysis. CPE is Constant Phase Element, which is usually used to fit equivalent circuit. The cycling performance of 6 M-Ni(OH)_2_ was further compared with that reported in the other relevant literatures (Figure 9f) [44,45,46,47,48,49,50]. It is clear that the synthesized 6 M-Ni(OH)_2_, subject to the longest cycle numbers, still exhibits an outstanding cycling stability when compared with those reported. 

### 3.4. Electrochemical Measurements of 6 M-Ni(OH)_2_//AC HSC Device

**Figure 10:**
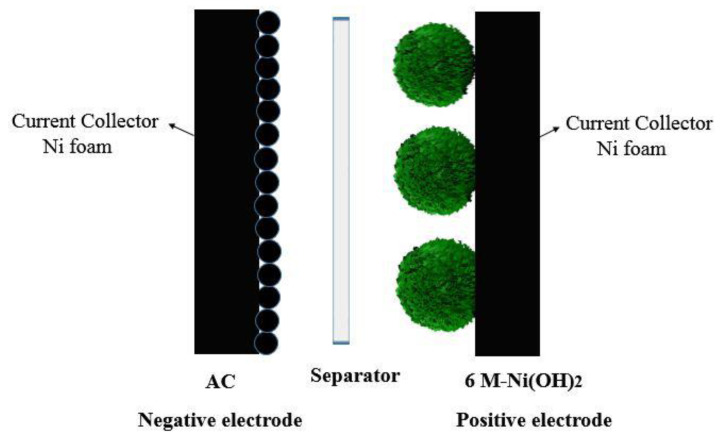
Schematic diagram of the 6 M-Ni(OH)_2_//AC hybrid supercapacitor (HSC).

Figure 10 displays the structure of the hybrid supercapacitor (HSC) device. The 6 M-Ni(OH)_2_ was used as the positive electrode owing to its excellent electrochemical properties. The AC electrode was used as the negative electrode on account of its high specific surface and outstanding electric conductivity, which is a typical characteristics of HSC devices. A piece of membrane was inserted in the medium as the separator to avoid the immediate contact of the two electrodes.

**Figure 11:**
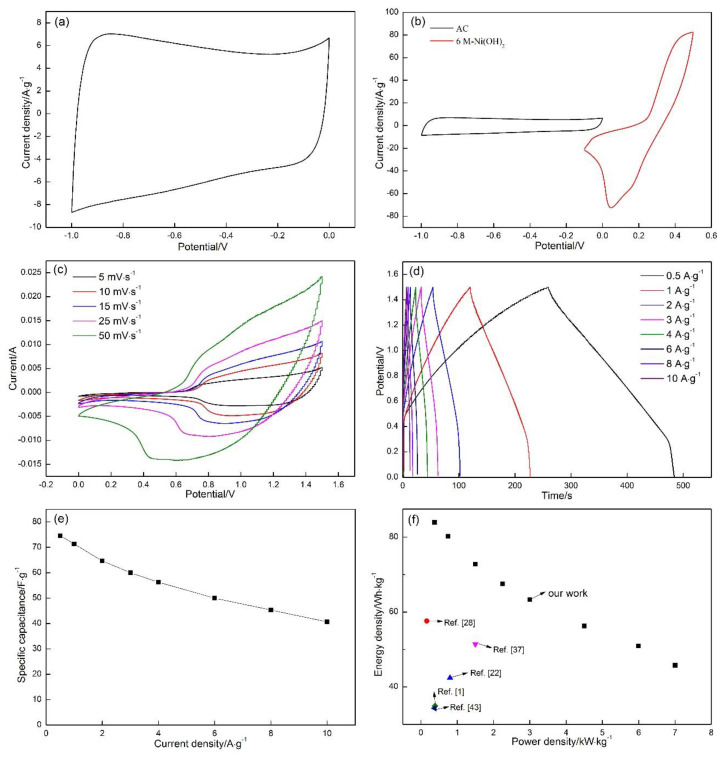
(**a**) CV curves of the active carbon (AC). (**b**) CV curves of the AC and 6 M-Ni(OH)_2_ electrodes at 50 mV·s^−1^. (**c**) CV curves of the HSC device tested at various scanning rates, of 5 mV·s^−1^ to 50 mV·s^−1^. (**d**) Galvanostatic charge-discharge tests (GCD) curves of the 6 M-Ni(OH)_2_//AC HSC. (**e**) Specific capacitance of the 6 M-Ni(OH)_2_//AC HSC at different current densities. (**f**) Ragone plot of the 6 M-Ni(OH)_2_//AC HSC.

Figure 11a demonstrates the CV curves of the AC in a three-electrode testing system with saturated calomel electrode (RE), a graphite sheet (CE), and the active material (WE), respectively. The 6 M KOH was selected as electrolyte. The CV curves of the sample display a typical rectangle-like shape confirming the EDLC (electrochemical double-layer) charge storage behaviors of the AC electrode materials [51]. The specific capacitance of the AC was then calculated, at about 115.78 F·g^−1^, according to the Equation (3). Moreover, the charge balance formula of q^+^ = q^−^ between two electrode materials should be followed to obtain excellent electrochemical properties [52], according to the Equation (5).

(5) $$\frac{m^{+}}{m^{-}}=\frac{C^{-}\times \Delta V^{-}}{C^{+}\times \Delta V^{+}}$$

In this formula, m^+^, ΔV^+^, and C^+^ refer to the mass, potential window, and specific capacity of the positive electrode, respectively. While m^−^, ΔV^−^, and C^−^ stand for the mass, potential window, and specific capacity of the negative electrode, respectively. The specific capacitance of 6 M-Ni(OH)_2_ was calculated to 597.99 F·g^−1^ (Table 3), while the specific capacitance of the AC was calculated to about 115.78 F·g^−1^, according to Equation (5), therefore the mass of the AC was finally calculated to 3.2 mg.

Figure 11b displays the potential window range of the HSC device. It can be observed that the potential windows of the AC and the Ni(OH)_2_ are of −1.0 V to 0 V and −0.1 V to 0.5 V, respectively. The potential window range of the HSC device results from the sum total of the two electrode materials. Meanwhile, with the scanning rate increasing from 5 mV·s^−1^ to 50 mV·s^−1^, the shapes of all curves are unchanged (Figure 11c). Thus, the working potential range of the device was finally determined to be 1.5 V.

In addition, GCD curves (Figure 11d) were also applied to further determine the specific capacitance of the HSC device. The specific capacitance values were calculated by the following formula [53]:

(6) $$C_{device}=\frac{I\Delta t}{m_{total}\Delta V}$$

where *C_device_* (F·g^−1^) is the specific capacitance of the HSC device, *I* (A) represents the constant discharge current, *m_total_* (g) is the mass of the whole samples, Δ*V* (V) is the potential window, and Δ*t* represents the discharge time. The values of the specific capacitance were calculated to be 74.6, 71.3, 64.7, 60.0, 56.3, 50.0, 45.3, 40.7 F·g^−1^ at the current densities of 0.5, 1, 2, 3, 4, 6, 8, 10 A·g^−1^, respectively. A total 54.5% of the specific capacitance was maintained with the current density increasing from 0.5 to 10 A·g^−1^ (Figure 11e), conforming the better rate capability of the HSC device.

The energy densities (*E*, Wh·kg^−1^) and power densities (*P*, W·kg^−1^) are another two significant parameters to evaluate the electrochemical performance of the device; according to the following formula [54]:

(7) $$E=\frac{1}{2}C\Delta V^{2}$$

(8) $$P=\frac{E}{\Delta t}$$

where *C* stands for the specific capacitance of the HSC device calculated by the total weight of the positive and negative active material. Δ*V* (V) and Δ*t* (s) represent the voltage window and discharge time, respectively.

The Ragone plot (Figure 11f) of the HSC device exhibits a high energy density of 83.925 Wh·kg^−1^ and a power density of 374.8 W·kg^−1^ at 0.5 A·g^−1^. With the current density increasing to 10 A·g^−1^, the energy density was 45.75 Wh·kg^−1^ and a power density of 7.5 kW·kg^−1^, which is much higher than those of the reported results. [1,27,33,42,49]

## 4. Conclusions

1. Sphere-like Ni-MOF with high specific surface area (67 m^2^·g^−1^) was fabricated by the hydrothermal method.

2. The concentrations of KOH played an important role in determining the morphology of the Ni(OH)_2._ The worm-like particles (Ni-MOF) transformed into fine nanosheets, resulting in the formation of a honeycomb-like structure (Ni(OH)_2_). Moreover, higher concentration caused the channels among nanosheets to greatly reduce in size, and made the spherical particles become denser.

3. The morphology of the Ni(OH)_2_ further effected the electrochemical properties of the electrode materials. The honeycomb-like 6 M-Ni(OH)_2_ exhibited a very high specific capacitance (1865 F·g^−1^ at 1 A·g^−1^, 1550 F·g^−1^ at 5 mV·s^−1^), and retained 59.46 % of its initial value at 10 A·g^−1^. While, 172% of its initial specific capacitance was retained after 20,000 cycles at 50 mV·s^−1^ in the three-electrode testing system.

4. The honeycomb-like 6 M-Ni(OH)_2_ also exhibited outstanding electrochemical properties in the two-electrode testing system. The as-prepared hybrid supercapacitor (HSC) device displayed superior electrochemical performance with a high energy density (83.925 Wh·kg^−1^ at a power density of 374.8 W·kg^−1^). All these results indicated that the honeycomb-like 6 M-Ni(OH)_2_ can be chosen as an excellent material for supercapacitors.

## Figures and Tables

**Table 1:** Pore size and specific surface areas of the Ni-MOF and Ni(OH)_2._

Samples	BET Surface Area (m^2^·g^−1^)	Total Pore Volume (cm^3^·g^−1^)	Average Pore Size (nm)
Ni-MOF	67	0.121	8.55
2 M-Ni(OH)_2_	130	0.447	13.62
6 M-Ni(OH)_2_	118	0.443	15.11
8 M-Ni(OH)_2_	98	0.390	15.87

**Table 2:** Specific capacitance of the samples at the same scanning rate of 5 mV·s^−1.^

Sample	2 M-Ni(OH)_2_	6 M-Ni(OH)_2_	8 M-Ni(OH)_2_	Ni-MOF
5 mV·s^−1^	875 F·g^−1^	1550 F·g^−1^	796 F·g^−1^	750 F·g^−1^

**Table 3:** Specific capacitance values of the 6 M-Ni(OH)_2_ samples at different scanning rates.

5 mV·s^−1^	10 mV·s^−1^	15 mV·s^−1^	25 mV·s^−1^	50 mV·s^−1^
1550 F·g^−1^	1069.27 F·g^−1^	875.86 F·g^−1^	649.22 F·g^−1^	597.99 F·g^−1^

**Table 4:** Specific capacitance values of the four samples at different current densities.

Current Density	Ni-MOF	2 M-Ni(OH)_2_	6 M-Ni(OH)_2_	8 M-Ni(OH)_2_
1 A·g^−1^	631 F·g^−1^	1380 F·g^−1^	1865 F·g^−1^	989 F·g^−1^
2 A·g^−1^	565 F·g^−1^	1187 F·g^−1^	1750 F·g^−1^	950 F·g^−1^
3 A·g^−1^	530 F·g^−1^	1024 F·g^−1^	1657 F·g^−1^	817 F·g^−1^
4 A·g^−1^	503 F g^−1^	895 F·g^−1^	1570 F·g^−1^	713 F·g^−1^
6 A·g^−1^	466 F·g^−1^	681 F·g^−1^	1418 F·g^−1^	562 F·g^−1^
8 A·g^−1^	437 F·g^−1^	487 F·g^−1^	1262 F·g^−1^	437 F·g^−1^
10 A·g^−1^	411 F·g^−1^	346 F·g^−1^	1109 F·g^−1^	334 F·g^−1^

**Table 5:** Parameters of the equivalent circuit model.

Sample	Rs (Ω)	Rct (Ω)	W_0_-R (Ω)
Ni-MOF	0.524	2.75	2.549
6M-Ni(OH)_2_	0.59	0.72	0.86
